# The cardiac care bridge program: design of a randomized trial of nurse-coordinated transitional care in older hospitalized cardiac patients at high risk of readmission and mortality

**DOI:** 10.1186/s12913-018-3301-9

**Published:** 2018-06-28

**Authors:** L. Verweij, P. Jepma, B. M. Buurman, C. H. M. Latour, R. H. H. Engelbert, G. ter Riet, F. Karapinar-Çarkit, S. Daliri, R. J. G. Peters, W. J. M. Scholte op Reimer

**Affiliations:** 1 ACHIEVE Center of Applied Research, Amsterdam University of Applied Sciences, Amsterdam, the Netherlands; 2 Department of Cardiology, Academic Medical Center, Amsterdam, the Netherlands; 3 Department of Internal Medicine, Section of Geriatric Medicine, Academic Medical Center, Amsterdam, the Netherlands; 4 Department of Rehabilitation, Academic Medical Center, Amsterdam, the Netherlands; 5 Department of General Practice, Academic Medical Center, Amsterdam, the Netherlands; 6 Department of Clinical Pharmacy, OLVG hospital, Amsterdam, the Netherlands

**Keywords:** Cardiology, Case management, Disease management, Transitional care, Rehabilitation

## Abstract

**Background:**

After hospitalization for cardiac disease, older patients are at high risk of readmission and death. Although geriatric conditions increase this risk, treatment of older cardiac patients is limited to the management of cardiac diseases. The aim of this study is to investigate if unplanned hospital readmission and mortality can be reduced by the Cardiac Care Bridge transitional care program (CCB program) that integrates case management, disease management and home-based cardiac rehabilitation.

**Methods:**

In a randomized trial on patient level, 500 eligible patients ≥ 70 years and at high risk of readmission and mortality will be enrolled in six hospitals in the Netherlands. Included patients will receive a Comprehensive Geriatric Assessment (CGA) at admission. Randomization with stratified blocks will be used with pre-stratification by study site and cognitive status based on the Mini-Mental State Examination (15–23 vs ≥ 24). Patients enrolled in the intervention group will receive a CGA-based integrated care plan, a face-to-face handover with the community care registered nurse (CCRN) before discharge and four home visits post-discharge. The CCRNs collaborate with physical therapists, who will perform home-based cardiac rehabilitation and with a pharmacist who advices the CCRNs in medication management The control group will receive care as usual.

The primary outcome is the incidence of first all-cause unplanned readmission or mortality within 6 months post-randomization. Secondary outcomes at three, six and 12 months after randomization are physical functioning, functional capacity, depression, anxiety, medication adherence, health-related quality of life, healthcare utilization and care giver burden.

**Discussion:**

This study will provide new knowledge on the effectiveness of the integration of geriatric and cardiac care.

**Trial registration:**

NTR6316. Date of registration: April 6, 2017.

**Electronic supplementary material:**

The online version of this article (10.1186/s12913-018-3301-9) contains supplementary material, which is available to authorized users.

## Background

Cardiac disease is the leading cause of hospitalization and mortality [1]. In the population of older hospitalized cardiac patients, 20% are readmitted and 10% die within 1 month post-discharge [2]. In addition to cardiac disease, geriatric conditions such as impaired activities of daily living (ADL) (77%), cognitive impairment (42%) and fall risk (30%) are highly prevalent [3]. The assessment of geriatric conditions is not currently part of routine medical evaluation in cardiology. As a result, these conditions are often unrecognized [4, 5] leading to an increased risk of new disabilities, readmission and death [3, 6].

The transition of care in which patients transfer between different settings increases the risk for adverse health outcomes due to inadequate attention to patients’ healthcare needs [7, 8]. For example, the failure to recognize geriatric conditions in older cardiac patients negatively impacts treatments post-discharge, e.g. because of nonadherence to (pharmacological) treatment in cognitively impaired patients [4] or poor participation in cardiac rehabilitation programs because of disabilities, the high intensity of these programs [9, 10], fatigue [11] and difficulties traveling to and from cardiac rehabilitation centers [12, 13]. This is unfortunate since cardiac rehabilitation has been shown to reduce cardiovascular risk factors, readmission and mortality in older cardiac patients [14].

Adequate guidance during hospitalization, during the transition from hospital to home and in the early post-discharge period may potentially reduce the risk of adverse events. Transitional care is a model that aims to continue care when patients transfer between different care settings, with a focus on patients’ needs [15, 16]. Recently, the Transitional Care Bridge program resulted in a 25% (HR 0.75, 95% CI 0.56–0.99, *P* = 0.045) reduction in mortality in acutely hospitalized older patients, by combining a Comprehensive Geriatric Assessment (CGA), an integrated care plan and a transitional care program, including visits during hospitalization and soon after discharge by a community care registered nurse (CCRN) [17]. However, with this case management approach no effects were found on readmission rates and ADL-functioning. We hypothesize that this may be caused by a main focus on case management within the care transition program with a lack of attention for disease management and rehabilitation after discharge.

The RESPONSE study of Jorstad et al. [18] involved a nurse-coordinated outpatient intervention that included guidance on lifestyle factors, biometric risk factors and therapy adherence in patients after an acute coronary syndrome. In this disease management approach, a relative risk reduction of 17.4% (*P* = 0.021) was found on the Systematic Coronary Risk Evaluation (SCORE), which is an integrated measure to estimate the risk of cardiovascular death in 10 years. In addition, a relative risk reduction of 34.8% (*P* = 0.023) was found on readmission.

Combining case management, disease management and home-based rehabilitation may have the potential to reduce readmission and mortality. Therefore, we developed the nurse-coordinated Cardiac Care Bridge transitional care program (CCB program) aiming to reduce unplanned hospital readmission and mortality in the first six months in comparison to usual care in older hospitalized cardiac patients at high risk of readmission and mortality. In this paper we report on the design of this program.

## Methods/Design

This study follows the Standard Protocol Items for Interventional Trials (SPIRIT) checklist (Additional file 1) [19]. The next paragraphs describe the Cardiac Care Bridge program, the study design and research methods.

### Design and setting

A single-blinded multi-center parallel group superiority trial with randomization at patient level will be performed in six hospitals in the Amsterdam region of the Netherlands: 1) Academic Medical Center (AMC), Amsterdam, 2) Amstelland Medical Center, Amstelveen, 3) BovenIJ Medical Center, Amsterdam, 4) Medical Center Slotervaart, Amsterdam, 5) Onze Lieve Vrouwe Gasthuis (OLVG), Amsterdam, 6) Tergooi Medical Center, Blaricum. In the transitional and post-clinical phase, five community nursing care organizations will participate: 1) Amstelring, 2) Buurtzorg Nederland, 3) Cordaan Home Care, 4) Evean, 5) Vivium Care Group. In the post-clinical phase, several community based physical therapists (PT) will participate. The recruitment for the study started on June 5, 2017 and will end after the last patient has been followed-up for 12 months, which is expected in December, 2019.

### Study population

Potential participants are all cardiac patients 70 years and older, acutely or electively admitted to the departments of cardiology or cardiothoracic surgery and admitted ≥ 48 h. They are eligible for inclusion if they are at high risk of functional decline according to screening instrument for frail elderly of the Dutch Safety Management Program (VMS instrument, Table 1). Four geriatric conditions (ADL, falls, malnutrition and delirium) are part of this screening. Oud et al. [20] also found a positive association between an increase of the number of risk factors with the VMS instrument and risk of death. Heim et al. [21] studied the optimal predictive value of frailty on adverse outcomes (death, functional decline and high healthcare use) with the VMS instrument. The strongest predictive value was found by a positive score on ≥ 3 risk factors in patients aged 70–79 and a positive score on ≥ 1 risk factor in patients aged ≥ 80 years. However, the screening of malnutrition may not be sensitive in cardiac patients because of an increased risk of weight gain due to decompensated heart failure [22]. Therefore, we considered patients aged 70–79 years with ≥ 2 risk factors and patients aged ≥ 80 years with ≥ 1 risk factor eligible for inclusion. In addition, patients at high risk of readmission and mortality are eligible to participate if they have had an unplanned hospital admission in the previous 6 months. This risk factor is associated with an increased risk of further readmissions and mortality [23, 24].

**Table 1:** Screening tool for vulnerable elderly of the Dutch Safety Management Program

Risk domain	Instrument	Questions	Cut-off	Score^a^
Fall risk	Single question	Did you fall in the last 6 months?	yes = 1	1
Malnutrition	SNAQ [53]	Assessing whether the patient: 1) lost weight unintentionally in the last 36 months and/or 2) experiences a decreased appetite and 3) used supplemental drinks or tube feeding	Question 1 = yes or Question 2 + 3 = yes	1
Delirium	Single questions	Assessing whether: 1) the patient has cognitive impairment; 2) the patient needed help with self-care in the last 24 h; 3) the patient has previously undergone a delirium	≥ 1 point = 1	1
ADL-functioning	KATZ-6 [54]	Assessing whether the patient needs help with: 1) bathing, 2) dressing, 3) toileting, 4) transferring from bed to a chair, 5) eating, and 6) whether the patient uses incontinence material	≥ 2 points = 1	1
Total score				0–4

*Abbreviations SNAQ* Short nutritional assessment questionnaire, *ADL* Functioning activities of daily living-functioning, *KATZ-6* Modified KATZ-6 index

^a^Patients are at high risk of functional decline if aged 70–79 years and score ≥ 2 or aged ≥ 80 years and score ≥ 1

Exclusion criteria are the following: 1) severe cognitive impairment, assessed with the Mini-Mental State Examination (MMSE < 15), 2) congenital heart disease, 3) terminal illness, defined as a life expectancy of less than 3 months as estimated by the treating physician, 4) transfer from or a planned discharge to a nursing home, 5) planned discharge to another department or another hospital not participating in this study, 6) inability to communicate in Dutch, 7) delirium as confirmed by patient’s physician and not resolved within 4 days after hospital admission.

### Randomization and blinding

After patients are screened for eligibility and have provided informed consent to a cardiac research nurse (CRN), the baseline assessment will be performed. After the baseline assessment patients will be randomized to the intervention or control group. Stratified block randomization (1:1) will be used with pre-stratification by study site and cognitive status based on the MMSE (15–23 vs ≥ 24). To ensure allocation concealment, a web-based data management program (Research Manager, http://deresearchmanager.nl/nl/home/) [25] and random permuted blocks of variable sizes will be used.

Group assignment will be blinded to patients. They will be informed that the study aim is to study different forms of post-discharge care and will receive only general information about the study protocol according to the postponed informed consent procedure of Boter et al. [26] Patients will be blinded to the aim of the intervention to prevent a potential Hawthorne effect [27, 28]. At the end of follow-up, patients (or their caregivers) will be fully informed about the content of the study intervention and the allocated treatment they received. Healthcare practitioners who execute the intervention cannot be blinded. Outcome assessments will be performed by research nurses who are blinded to the allocated treatment. Statistical analyses will be performed according to a predefined statistical analysis plan (see Statistical Analysis paragraph) by investigators blinded to group assignment.

Due to the minimal expected side effects related to the intervention of the CCB care program a data monitoring committee is not mandatory for this trial.

### Hospital care for all included patients

Table 2 shows the time frame and components of the CCB program in the intervention and control groups. All included patients will receive a CGA within 72 h after admission by a CRN, which will also serve as the baseline study measurement (Table 3). The CGA identifies health issues in the somatic, psychological, social and functional domains, including problems related to polypharmacy, malnutrition, fall risk, delirium, depression and quality of life. Cardiovascular risk factors (e.g. body mass index, smoking, alcohol use and physical performance) will also be assessed. Following assessment, consenting patients will be randomized to the intervention or control group.

**Table 2:** Time frame and components of the Cardiac Care Bridge program and the control group

Time Frame	Intervention component	Baseline – outcome measures	Professionals involved	Intervention	Control
Clinical phase					
≤ 72 h after hospital admission	*CGA* ^a^	Baseline	CRN^b^	X	X
≤ 72 h after hospital admission	Integrated care plan		CRN^b^	X	
During hospital stay	Geriatric team consultation in case of ≥ 5 identified health issues or ≥ 1 psychological issue		CRN^b^, CNS^c^, geriatrician	X	
Discharge phase					
Before hospital discharge	In-person handover of the CGA^a^, integrated care plan and medical treatment plan		CRN^b^, CCRN^d^	X	
Before hospital discharge	Visit of CCRN^d^ to participant		CCRN^d^	X	
At discharge	Medical discharge letter		Cardiologist, GP^e^, CCRN^d^	X	X
Post-clinical phase					
≤ 2 days after hospital discharge	Home visit 1. Medication reconciliation and integrated care plan		CCRN^d^	X	
≤ 1 week	*Home visit 2. Intake home based cardiac rehabilitation and integrated care plan*		CCRN^d^, PT^f^	X	
Week 1	*Two home-based cardiac rehabilitation sessions*		PT^f^	X	
Week 2	*Two home-based cardiac rehabilitation sessions*		PT^f^	X	
Week 3	Home visit 3. lifestyle promotion and self-management		CCRN^d^ PT^f^	X X	
	*Two home-based cardiac rehabilitation sessions*		PT^f^	X	
Week 4	*Two home-based cardiac rehabilitation sessions*		PT^f^	X	
Week 5	*Two home-based cardiac rehabilitation sessions*				
Week 6	Home visit 4. Evaluation of integrated care plan and home-based cardiac rehabilitation		CCRN^d^ PT^f^	X X	
	*Two home-based cardiac rehabilitation sessions*				
≤ 12 weeks	Home visit 5. If indicated by the CCRN^b^				
3 months		Follow-up telephone	Research Nurse	X	X
6 months		Follow-up home visit	Research Nurse	X	X
12 months		Follow-up telephone	Research Nurse	X	X

^a^Comprehensive Geriatric Assessment (CGA)

^b^Cardiac Research Nurse (CRN)

^c^Clinical Nurse Specialist in geriatrics (CNS)

^d^Community Care Registered Nurse (CCRN)

^e^General Practitioner (GP)

^f^Physical therapist (PT)

**Table 3:** Baseline assessment, outcome measures and time points in the Cardiac Care Bridge

	CGA	Question or instrument	T0*	T0 + ^†^	T1^‡^	T2^§^	T3^||^
Sociodemographic data							
Age		Date of birth	X^¶^				
Gender			X^¶^				
Postal code			X				
Living arrangement			X				
Marital status			X				
Ethnicity		Patients’ country of birth	X				
Education			X				
Mortality		Date of death	X^¶^		X^¶^	X^¶^	X^¶^
Medical data							
Diagnosis (and history) of cardiac disease			X^¶^				
Comorbidities		CCI [55]	X^¶^				
Date of hospitalization			X^¶^				
Hospitalization department			X				
Functional domain							
ADL- and iADL-functioning	+	ALDS [35]	X		X	X	X
Functional status		Specific Activity Scale [33]	X			X	
Hearing impairment	+	Do you experience difficulties with hearing, despite the use of a hearing aid?	X				
Visual impairment	+	Do you experience difficulties with your vision, despite the use of glasses?	X				
Fatigue	+	NRS	X			X	
Falls	+	Frequency	X		X	X	X
Fear of falling	+	NRS	X		X	X	X
Physical domain							
Nutritional status	+	SNAQ [53]	X		X	X	X
Pain	+	NRS [56]	X			X	
Dizziness	+	Do you currently suffer from dizziness If yes, does this affect your daily living?	X			X	
Shortness of breath	+	Do you currently suffer from shortness of breath? If yes, does this affect your daily living?	X			X	
Angina pectoris	+	Do you currently suffer from angina pectoris If yes, does this affect your daily living?	X			X	
Heart palpitations	+	Do you currently suffer from heart palpitations? If yes, does this affect your daily living?	X			X	
Incontinence	+	Do you suffer from incontinence? If yes, do you suffer from incontinence of urine and/or defecation?	X			X	
Presence of urinary catheter	+	Do you have a urinary catheter? If yes, did you have the urinary catheter before hospitalization?	X			X	
Nycturia	+	Do you currently suffer from nycturia? If yes, does this affect your daily living?	X			X	
Handgrip strength	+	Jamar [57]	X			X	
Psychological domain							
Cognitive status	+	MMSE [58]	X			X	
Depression & apathy	+	GDS-15[41]	X			X	
Anxiety	+	HADS-A [38]	X		X	X	X
Quality of life	+	EQ-5D-5 L [40]	X		X	X	X
Smoking status		Do you smoke or did you smoke in the past? If yes, how many cigarettes per day and for how many years?	X		X	X	X
Alcohol use		AUDIT-C [59]	X		X	X	X
Social domain							
Caregiver burden		TOPIC-MDS [41]	X			X	X
Medication use							
Polypharmacy	+	Do you use five or more different medications?	X			X	
Medication adherence	+	Medication Adherence Questionnaire	X		X	X	X
Side effect of medication	+	Do you experience difficulties or side effects with medication use?	X			X	
Type of medication		Type, frequency and dose of medication	X^¶^		X^¶^	X^¶^	X^¶^
Physical performance							
Physical performance		30-s chair stand test [60]		X		X	
Mobility		SPPB [36]	X			X	
Physical capacity		2 MST [37]	X	X		X	
Perceived exertion		Borg RPE scale [61]	X	X		X	
Dyspnoea		MRC dyspnoea scale [62]		X		X	
Parameters							
BMI		Weight and length	X			X	
Waist circumference			X			X	
Blood pressure		mmHg	X			X	
Heart frequency		BPM	X			X	
Respiratory rate			X			X	
Blood parameters		Hemoglobin	X^¶^		X^¶^	X^¶^	X^¶^
		Albumin	X^¶^		X^¶^	X^¶^	X^¶^
		Creatinine	X^¶^		X^¶^	X^¶^	X^¶^
		Total cholesterol	X^¶^		X^¶^	X^¶^	X^¶^
		LDL-cholesterol	X^¶^		X^¶^	X^¶^	X^¶^
		HDL-cholesterol	X^¶^		X^¶^	X^¶^	X^¶^
		Triglyceride	X^¶^		X^¶^	X^¶^	X^¶^
		Glucose / HbA1C	X^¶^		X^¶^	X^¶^	X^¶^
Healthcare utilization		TOPIC-MDS [41]					
Readmission		Have you been hospitalized in the last six months? If yes, what was the hospitalization diagnosis and in what hospital were you readmitted?			X^¶^	X^¶^	X^¶^
Emergency visits		Have you visited the emergency or cardiac emergency room in the last six months? If yes, how many times and for what reason?			X*	X*	X*
Nursing home admission		Have you been admitted to a nursing home in the last months? If yes, for how many weeks?			X	X	X
General practice consult		Have you had a consult with your general practitioner in the last month? If yes, was this during office hours or during the evening, night or weekend and how many times in total?			X	X	X
Home visit of GP		Have you had a home visit from your GP in last month? If yes, was this during office hours or during the evening, night or weekend, and how many times in total?			X	X	X
Home care		Do you receive home care? If yes, is this care assistance and/or domestic help, and how many hours per week?			X	X	X
Day care		Do you have day care? If yes, how many days per week?			X	X	X
Cardiac rehabilitation use		Do you participate in cardiac rehabilitation in a rehabilitation center or outpatient clinic?			X	X	X
Physical therapy		Do you participate in cardiac rehabilitation in a rehabilitation center or outpatient clinic?			X	X	X

*Abbreviations CCI* Charlson comorbidity index, *ALDS* Amsterdam linear disability scale*, NRS* numeric rating scale, *SNAQ* short nutritional assessment questionnaire, *MMSE* mini mental state examination, *GDS-15* geriatric depression Scale-15, *HADS-A* hospital anxiety and depression scale-anxiety subscale*,* EuroQol-5D Euroqol quality of life, *MDS* minimal dataset, *SPPB* short physical performance battery, *2MST* 2 Minute step test, *Borg RPE scale* ratings of perceived exertion scale, *MRC Dyspnea Scale* Medical Research Council dyspnea scale, *mmHg* millimetre of mercury, *BPM* beats per minute

*T0: baseline, ≤ 48 h after admission; ^†^T0+: within 2 weeks after hospitalization during home-based cardiac rehabilitation intake; ^‡^T1: 3 months after hospitalization, follow-up by telephone; ^§^T2: 6 months after hospitalization, follow-up by home visit; ^||^T3: 12 months after hospitalization, follow-up by telephone. ^¶^Data will be obtained from the medical record

### Intervention

The CCB program encompasses three phases of the care process: 1) clinical phase, 2) discharge phase from hospital to home and 3) post-clinical phase after hospital discharge. The intervention consists of three components: 1) case management, 2) disease management and 3) home-based cardiac rehabilitation. Medication management is an important topic in the three phases of the CCB intervention and is part of all three components.

### Phase 1: Clinical phase

Patients randomized to the intervention group will receive an integrated care plan based on geriatric and cardiac conditions identified by the CGA. This plan will be developed by the CRN together with the patient as follows. The CRN discusses identified health issues, asks if the patient recognizes them and what issues they prioritize for treatment. The integrated care plan is used to prioritize care during the three phases of the intervention. In case of ≥ 1 health issue in the psychological domain or ≥ 5 potential health issues in total, the geriatrician will be consulted. If indicated, the CRN also consults with other disciplines.

### Phase 2: Discharge phase

At least one day before discharge, the CCRN visits the patients to discuss and prepare discharge to home. A personalized face-to-face handover between the CRN and the CCRN is completed using a standardized discharge checklist. In case of logistical difficulties the handover is performed by video call via tablet. The CGA, integrated care plan and ongoing interventions are discussed. In addition, the current medical condition, medication prescriptions and therapy advices a patient needs to adhere to (e.g. fluid restrictions in case of heart failure) are discussed. Finally, the CRN contacts the primary care PT by telephone to arrange home-based cardiac rehabilitation.

### Phase 3: Post-clinical phase

After discharge home, the CCRN and PT continue care at home. The focus of these visits is in the first month post-discharge since this is when patients are at highest risk for readmission, mortality and functional decline [2, 3]. The CCRN visits the patient four times post-discharge; within 2 days, at 1, 3 and 6 weeks and if needed one more visit within 12 weeks post-discharge. During all home visits, the CGA, the integrated care plan and patients’ current medical condition is evaluated. During the first home visit medication reconciliation is performed by the CCRN to obtain the most accurate possible list of a patient’s current medications [29, 30]. This is done by comparing all the medications that the patient is taking (including over-the-counter drugs, herbals and vitamins) to those listed in the provided medication records (medication overview from the community pharmacy and the discharge summary from the hospital). Within 48 h after discharge the discharge summary, which contains an overview of the medications at discharge, reasons for changes in medication and results of diagnostic tests is sent from the hospital to the CCRN and pharmacist who is part of the research team.

In Table 2, the home visit schedule is presented, including specific themes during the home visits. The CCRN is allowed to deviate from the home visit schedule if indicated, for example because of changes in patients’ health status. During the home visits, the CCRN will indicate and refer if there is a need for additional care (domiciliary or otherwise) during or after the intervention period. For specific questions related to patients’ health status or medication discrepancies identified during medication reconciliation, the CCRN has access to the cardiac team of the hospital, the general practitioner (GP), pharmacist according to local communication routes or protocols of the hospitals. During the home visits the CCRN observes signs and symptoms of actual or potential drug-related problems (DRP), such as side-effects and inappropriate medication use (e.g. nonadherence) by using a recently developed instrument (Additional file 2. Adapted Red Flag instrument) based on the Red Flag instrument by Sino et al. [31] The observed problems are documented by the CCRN in the Adapted Red Flag instrument and evaluated by the pharmacist-investigator who has identified DRP and proposed suitable solutions. Subsequently the CCRN discusses these DRP and proposed solutions with the responsible healthcare providers.

The PT provides two home-based cardiac rehabilitation sessions per week during the first 6 weeks post-discharge. This program is based on therapy advices according to the Dutch multidisciplinary guideline of cardiac rehabilitation [32]. Depending on the patient’s functional status a stepwise graded exercise approach will be followed, starting with low intensity functional rehabilitation (class IV or higher on the Specific Activity Scale [33]) to the Metabolic Equivalent of Task level [34] (MET-level) needed for their goals and desired activities, as described in the rehabilitation plan. Exercise therapy will be adapted to comorbid diseases according to current guidelines. Within the last 2 weeks of the rehabilitation program, patient’s functional status will be evaluated. The CCRN and PT work in close collaboration during the intervention to tailor care and to evaluate progress. They have a joint home visit in the first week after discharge to verify and agree on the integrated care plan in relation to patients’ priorities.

In case of readmissions to participating hospitals and wards during the study follow-up of 12 months, patients will repeatedly receive the CCB program with exception of the rehabilitation exercise component. This is due to the limit on physical therapy sessions funded by Dutch healthcare insurance policies.

### Usual care

Patients in the control group will receive usual care during hospitalization and after discharge. During hospitalization, other disciplines are consulted as needed. The control group may receive geriatric care if the patients’ treating physician consults the geriatric team. All participating hospitals have a geriatric consultation team that can be consulted by the patients’ treating physician on indication. After discharge, care as usual may include medical care by a cardiologist according to the national cardiovascular guidelines and a cardiac nurse specialist, if available. Also, control group patients can be referred to center-based cardiac rehabilitation. According to the Dutch multidisciplinary guideline of cardiac rehabilitation, center-based cardiac rehabilitation consists two one-hour exercise sessions per week during 6 weeks [32]. However, it is expected that only a small number of patients in the control group will receive center-based cardiac rehabilitation due to their age, illness and clinical complexity.

Standard primary care will be provided in both the intervention and the control group. For non-cardiovascular problems, the GP is the primary healthcare provider. Optional care provision in the GP practice includes secondary prevention, medication titration, regular evaluations of physical health status and referral to other disciplines. In both groups the GP will be informed about the hospitalization by a discharge letter from the medical specialist. In the intervention group the GP is informed about the patients’ study participation by letter. During the intervention, the CCRN will be an extra liaison between care providers in case of medical, mental or social issues.

In the Netherlands virtually all citizens have basic healthcare insurance, which includes coverage of primary care visits, hospital outpatient visits, hospitalizations and prescribed medication. Dutch citizens can also purchase optional supplementary insurance, which includes physical therapy and other services.

### Training for healthcare providers and implementation

The CCB program combines case management, disease management and home-based cardiac rehabilitation, which require additional skills of healthcare providers. The participating CRNs and CCRNs will therefore follow a 5-day training program focussing on case management and disease management which addresses geriatric conditions, the performance of the CGA, development of an integrated care plan, pathophysiology of common cardiac diseases, early detection of physical deterioration and complications, pharmaceutical treatments and cardiac rehabilitation, including lifestyle counselling [9–13]. The participating PTs followed 2,5 day of the 5-day training program together with the CRNs and CCRNs, focussing on pathophysiology of common cardiac diseases, early detection of physical deterioration and complications, pharmaceutical treatments and cardiac rehabilitation, including lifestyle counselling.

We performed a feasibility process in six participating hospitals from June 2016 until May 2017 to check for potential inclusion rates to implement the study protocol and to train CRNs in data collection. In total 45 patients were included in this pilot phase. After successful implementation, we started the official inclusion stepwise per hospital with the first hospitals starting in June 2017.

### Sample size calculation

**Fig. 1:**
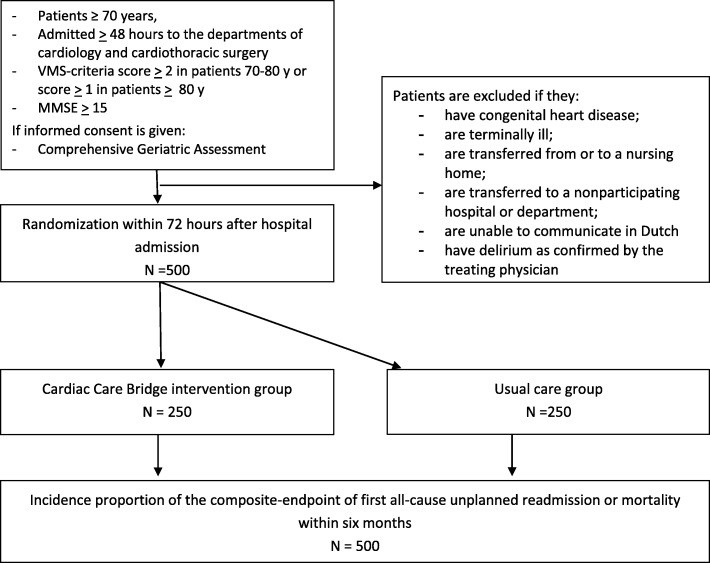
Flowchart of patient selection and randomization

The sample size calculation is based on findings in a relevant subpopulation (101/674) of cardiac patients of the Transitional Care Bridge program [17], a comparable study including hospitalized patients ≥ 65 years at high risk of functional decline. Based on a six-month incidence rate of 44% (readmission and mortality combined) in the usual care subpopulation of the Transitional Care Bridge program and a minimal important difference of 12.5% in absolute risk reduction (from 44 to 31.5%) in patients in the intervention arm, (2-sided alpha of 0.05; power of 80%), a sample size of 235 patients per group is required. To compensate for an assumed 5% loss to follow-up, the total sample size per group will be 250 (Fig. 1).

### Outcomes and measurements

#### Primary outcome

The primary outcome is the incidence of first all-cause unplanned readmission or mortality within 6 months post-randomization.

#### Secondary outcomes

Secondary outcomes will be measured at three, 6 and 12 months. Data will be collected by telephone at three and 12 months and at 6 months by a home visit of a blinded research nurse. Table 3 provides an overview of the data collection on different time points. The secondary outcomes are the following:

- The incidence of the first all-cause unplanned hospital readmission or mortality within 3 months and 12 months after randomization (triangulated by self-reporting and hospital data management system)
- Activities of Daily Living (ADL)- / instrumental ADL-functioning at 3*,* 6 and 12 months after randomization (the AMC Linear Disability Score) [35]
- Functional capacity at 6 months after randomization (Short Physical Performance Battery [36] and 2-min step test [37])
- Medication adherence (questionnaire and pharmacy dispensing records) at 3*,* 6 and 12 months after andomization
- Anxiety and depression at 6 months after randomization (HADS-anxiety [38] and Geriatric Depression Scale-15 [39])
- Health-related quality of life at 6 and 12 month after randomization (EuroQol-5D-5 L) [40]
- Healthcare utilization at 3*,* 6 and 12 months after randomization (extension of *The Older Persons and Informal Caregivers Survey - Minimum Data Set (TOPIC-MDS)* [41] including readmission, emergency visits, GP visits, physical therapy and cardiac rehabilitation)
- Caregiver burden, at 6 and 12 months after randomization (TOPIC-MDS) [41]

### Statistical analyses

All analyses will be performed according to a predefined statistical analysis plan, which is published in the Netherlands Trial Register (NTR6316). The primary analyses will be performed according to the intention-to-treat principle. Outcomes will be reported as unadjusted risk differences and their 95% confidence intervals. Adjusted analyses using multivariable logistic or linear regression models, as appropriate, will focus on the incidence proportion of the composite endpoint of readmission and mortality up to 6 months. All analyses will be adjusted for the following potential confounders: age, sex, Charlson Comorbidity Score, MMSE, cardiovascular diagnosis, length of stay and living arrangement. In addition, subgroup analyses will be performed for cardiac diagnosis, frailty status with the VMS screening tool, cognitive status with the MMSE and social economic status. Data will be collected by an electronic Case Record Form in Research Manager [25], a web-based data management program. Multiple imputation will be used as a sensitivity analysis to assess the impact of missing values.

### Cost effectiveness analysis

We will perform a cost-effectiveness analysis from a societal perspective. Incremental cost-effectiveness ratios (ICERs) will be calculated by dividing the difference in total costs between the intervention group and the control care group by difference in readmission/mortality rates and Quality Adjusted Life Years (QALYs). The uncertainty surrounding the ICERS will be estimated with non-parametric bootstrapping (5000 replications). The intention to treat principle will be applied to analyse the data. Missing values for cost and effect data will be predicted by multiple imputation.

### Process evaluation

Quantitative data will be collected by using pre-defined process indicators to measure study performance and adherence to the intervention by the patient, CRN, CCRN and PT. Process indicators will be used to study fidelity and adherence to the study protocol. Process indicators are focussed on documentation, communication between healthcare providers, consultation of disciplines, referral to healthcare providers and medication issues. All process indicators will be quantified by nominator and denominator and collected through existing resources. Usual care will be documented to be able to assess the difference between the intervention and control group. In addition, qualitative data will be collected during the intervention by focus groups with healthcare providers and in semi-structured interviews with patients and informal caregivers to evaluate satisfaction with the intervention. These data will be analysed to identify factors that promote or impede future implementation of the CCB care program.

### (Serious) adverse events

Study related adverse events (AE) will be reported when the AE occurs during the comprehensive geriatric assessment and baseline data collection or after discharge when the AE occurs during the home visits by the CCRN or during the physical therapy sessions / self-practice physical therapy sessions by the patients within the intervention period (till 12 weeks post-discharge). After 12 weeks, the intervention has stopped. Therefore, serious adverse events after this period are not expected to be caused by the study and will only be recorded during the annual security reports.

## Discussion

This protocol for a multi-center randomized controlled trial is designed to prevent hospital readmission and mortality after hospitalization in cardiac patients ≥70 years old who have been admitted to the department of cardiology or cardiothoracic surgery. Older patients who are discharged after hospitalization for a cardiac disease are at high risk of adverse outcomes, in particular early readmission and mortality [42, 43]. This vulnerable patient population is currently underrepresented in medical research, resulting in a lack of evidence on how to improve their outcomes [44–46].

In this paper we describe the study protocol of the CCB care program in which we combine three care components: case management, disease management and home-based cardiac rehabilitation that will be provided during and after hospitalization for cardiac disease. Multidisciplinary collaboration between the in-hospital cardiac team, including the CRN and the cardiologist, the clinical nurse specialist in geriatrics and the pharmacist, CCRN and PT in primary care, is an important part of the study intervention. By introducing face-to-face (‘warm’) handovers before discharge and a joint home visit of the CCRN and PT and support from a pharmacist, we expect to reduce information loss, improve the continuity of treatment, leading to a decrease in readmission and mortality.

Current literature on transitional care and cardiac rehabilitation in older high risk patients focuses mainly on the separate components of case management, disease management and home-based cardiac rehabilitation. In the recent Transitional Care Bridge program, a nurse-coordinated transitional intervention in acutely hospitalized high-risk older patients led to a 25% reduction in mortality, HR 0.75; 95% CI 0.56–0.99. However, there was less impact on time to first hospitalization, HR 1.21; 95% CI 0.91–1.60 [17]. The RESPONSE trial, a nurse-coordinated disease management intervention after a coronary syndrome led to a 35% reduction in readmission rates and 17.5% reduction in cardiovascular risk factors in a general cardiac patient population aged < 80 years [18]. Studies on cardiac rehabilitation in the elderly found positive trends on patients’ functional ability [9, 47]. However, most of these were pilot studies with limited power. In addition to the heterogeneity of the study effects of these studies, the components do not fully meet patients’ needs in the care continuum [48]. Therefore, we expect that a combination of care components focusing on patients’ needs has a greater likelihood of being effective. The Korinna trial [49] combined both case management and disease management in older patients after a myocardial infarction, but did not find a relevant effect on hospital readmission (HR 1.01; 95% CI 0.72–1.41). Compared to the intervention in the Korinna trial [49], the CCB program is focussed on a broader cardiac patient population instead of patients after acute myocardial infarction only. Other differences are the emphasis of the CCB program on the first period after hospitaization with a first home visit within 2 days after discharge and the additional home based cardiac rehabilitation program.

### Strengths and limitations

The first strength of this study is that it includes a wider variety of the cardiac patient population than previous studies. This is because it selects patients based on their risk of readmission and mortality, instead of diagnosis, and because it selects from six hospitals in both an urban and a rural area. Second, this study has a robust design and includes a postponed informed consent procedure, which assures high internal validity. Third, a comprehensive geriatric assessment is used to develop a personalized care plan, including cardiac and geriatric care, that is transferrable across settings and healthcare providers. Fourth, due to the comprehensive nature of the intervention, it will not be possible to evaluate separate intervention components on their effectiveness but by use of process indicators we will collect data on the execution of the components of the intervention and performance of the involved healthcare providers to support interpretation of the study results. Finally, the intervention has been designed in multi-disciplinary collaboration between nurses, physical therapists, pharmacists and physicians.

This study also has some limitations. First, we exclude patients with delirium and dementia. These patients are at risk for readmission [50] and mortality [51, 52] and therefore could potentially benefit from this intervention. However, it is not possible to include these patients in the CCB program because of ethical considerations. Secondly, the face-to-face handover between de CRN and CCRN is a promising intervention but also challenging due to logistical difficulties as, for example, the sometimes unpredictable discharges from the hospital. An alternative handover was introduced by video call via tablets.

In summary, the CCB program aims to significantly reduce the primary composite endpoint of unplanned hospital readmission and mortality in older cardiac patients.

## Additional files

Additional file 1: Standard Protocol Items Recommendations for Interventional Trials (SPIRIT) Checklist of the Cardiac Care Bridge program study protocol. (DOC 121 kb)

Additional file 2: Adapted Red Flag Instrument. Adapted version of the Red Flag Instrument by Sino et al. [33]. (DOCX 39 kb)

## Abbreviations

*2MST*: 2 Minute Step Test
ADL-functioning: Activities of Daily Living-functioning
ALDS: Amsterdam Linear Disability Scale
Borg RPE scale: Ratings of Perceived Exertion scale
BPM: Beats per minute
*CCI*: Charlson Comorbidity Index
CCRN: Community Care Registered Nurse
CGA: Comprehensive Geriatric Assessment
CNS: Clinical Nurse Specialist in geriatrics
CRN: Cardiac Research Nurse
EuroQol-5D: Euroqol quality of life
*GDS-15*: Geriatric Depression Scale-15
GP: General Practitioner
HADS-A: Hospital Anxiety and Depression Scale-Anxiety subscale
KATZ-6: Modified KATZ-6 index
MDS: Minimal Dataset
MmHg: Millimetre of mercury
*MMSE*: Mini Mental State Examination
MRC Dyspnea Scale: Medical Research Council Dyspnea Scale
*NRS*: Numeric Rating Scale
PT: Physical therapist
SBSQ-D: Set of Brief Screening Questions – Dutch
*SNAQ*: Short Nutritional Assessment Questionnaire
*SPPB*: Short Physical Performance Battery
