# Supplementary material for: The cardiac care bridge program: design of a randomized trial of nurse-coordinated transitional care in older hospitalized cardiac patients at high risk of readmission and mortality
Source: BMC Health Serv Res. 2018 Jun 28;18:508. doi: 10.1186/s12913-018-3301-9 (PMC6025727; doi:10.1186/s12913-018-3301-9)
Supplement: Supplementary file 2 — Adapted Red Flag Instrument. Adapted version of the Red Flag Instrument by Sino et al. [33]. (DOCX 39 kb) [file 12913_2018_3301_MOESM2_ESM.docx]

Does the patient currently experiences one of the symptoms listed below? If the answer is yes, write YES in column ‘SYMPTOM PRESENT’ and ask the following questions: ‘Did the symptom appear suddenly?’ **(YES/NO)**, ‘Is the symptom acceptable/not bothersome?‘ **(YES/NO)** and ‘**Does** the patient think the symptom is caused by medication?’ **(YES/NO)**. If yes, write down the name of the medication. If the patient has a symptom which is not listed below, write the symptom down in the row ‘Other symptom’.

**CAUTION: Always call 112 in case of a sudden onset of a symptom**

|  | **SYMPTOM PRESENT?** | **SUDDEN?** | **ACCEPTABLE?** | **NAME MEDICINE?** |
| --- | --- | --- | --- | --- |
| **Cardiology** |  | | | |
| - Tightness of chest |  |  |  |  |
| - Extreme high/low* blood pressure compared to normal |  |  |  |  |
| - Weight gain of 2 kg or more in 2-3 days and/or increased swelling of the legs, ankles, abdomen* |  |  |  |  |
| - (Exacerbation of) shortness of breath/ waking up in the night, suddenly breathless * |  |  |  |  |
| - Sudden rapid/irregular* heartbeat |  |  |  |  |
| - Dizziness when standing up |  |  |  |  |
| - Red-glossy and/or painful legs (Deep venous thrombosis) |  |  |  |  |
| **Bleedings** |  | | | |
| - Black stool color |  |  |  |  |
| - Easy bruising/repeated episodes of nosebleeds * |  |  |  |  |
| **Neurology** |  | | | |
| - Recently fainted |  |  |  |  |
| - Paralysis (facial / on one side of the body and difficulty with speaking |  |  |  |  |
| - Confusion (delirium) |  |  |  |  |
| - Altered level of consciousness (drowsy) |  |  |  |  |
| - Frequent headaches |  |  |  |  |
| **Gastrointestinal disorders,** |  | | | |
| - No bowel movement in 5 days |  |  |  |  |
| - Nausea, vomiting and/or loss of appetite* |  |  |  |  |
| **-** Acid reflux |  |  |  |  |
| - Stomach ache |  |  |  |  |
| **Other** |  | | | |
| - Fatigue (listlessness) |  |  |  |  |
| - Excessive thirst |  |  |  |  |
| - Dry mouth and/or decreased urinary frequency compared to normal * |  |  |  |  |
| - Severe muscle ache |  |  |  |  |
| - Dry and hacking cough |  |  |  |  |
| - Other symptom, such as: |  |  |  |  |

*Circle the applicable answer.

Does the patient have any problems with medication use, medication adherence and/or adjusting the medication regimen to the daily schedule? Observe and assess problems with medication use by asking the questions listed below. Please tick the box “YES” if applicable. Additional comments concerning a symptom or problems with medication use can be specified in the comments field.

| **ASSESSMENT OF MEDICATION MANAGEMENT** | **YES?** |
| --- | --- |
| - The patient keeps old (unused) medication around *(e.g. because multi-dose drug dispensing is not adjusted with changed medication)*  - The patient has medication from previous days in the pill box or multi-dose drug dispensing | O  O |
| - The patient does not store medication properly *(e.g. medication is stored in different places and/or different containers)*  - The patient uses expired medication *(e.g. due to functional illiteracy expiration or vision problems)*  - The patient does not store medication in the original containers and/or at the recommended storage conditions *(e.g. cool, dry, dark)* | O  O  O |

| **QUESTIONS MEDICATION USE** | **YES?** |
| --- | --- |
| - Does the patient have difficulty with ordering medication and therefor regularly runs out of medication?  - Does the patient have trouble telling mediation apart? *(e.g. when using multiple medication)*  - Does the patient experiences difficulty with adjusting the medication regimen to the daily schedule? - Does the patient experiences problems with reading and/or understanding the instructions for use? *(e.g. due to functional illiteracy or vision problems)* - Does the patient experiences difficulty with handling the immediate packaging and pressing the medication out? - Does the patient experiences difficulty with completing preparation of medication before use and administration? *(e.g. administration of insulin, inhalation and anti-coagulant medication, applying medication patches and eye ointment, or instilling eye drops and ear drops)* - Does the patient encounter difficulty with taking medication? *(e.g. lodging of medication in the mouth or throat, problems with the flavor of medication, or no motivation to take medication)* | O  O  O  O  O  O  O |
| - Does the patient drink more than 3 glasses of alcohol a day? | O |

| **QUESTIONS MEDICATION ADHERENCE** |
| --- |
| “Almost everyone occasionally misses one or more doses of their medicines. Each person has its own way of taking medication. Sometimes this can deviate from the doctor’s prescription. I would like to ask you some questions regarding your medication intake. There is no right or wrong answer.”   - **From the moment you were admitted to the hospital for your heart, which medicine(s) did you forget to take?**   Explanation:_____________________________________________________________________________________________________________________________________________________________________________________________________________   - **From the moment you were admitted to the hospital for your heart, how often did you forget to take the medicine(s)?**   Explanation:____________________________________________________________________________________________________________________________________________________________________________________________________________   - **From the moment you were admitted to the hospital for your heart, which medicine(s) did you consciously not take as prescribed by the doctor? (e.g. more, less, skipped, stopped)**   Explanation:_________________________________________________________________________________________________ |

| **COMMENTS** |
| --- |
|  |

*Circle the applicable answer.
